# Supplementary material for: Timed chromatin invasion during mitosis governs prototype foamy virus integration site selection and infectivity
Source: Nucleic Acids Res. 2025 May 31;53(10):gkaf449. doi: 10.1093/nar/gkaf449 (PMC12125541; doi:10.1093/nar/gkaf449)
Supplement: gkaf449_Supplemental_Files [file gkaf449_supplemental_files.zip › supplementary_figures.pdf]

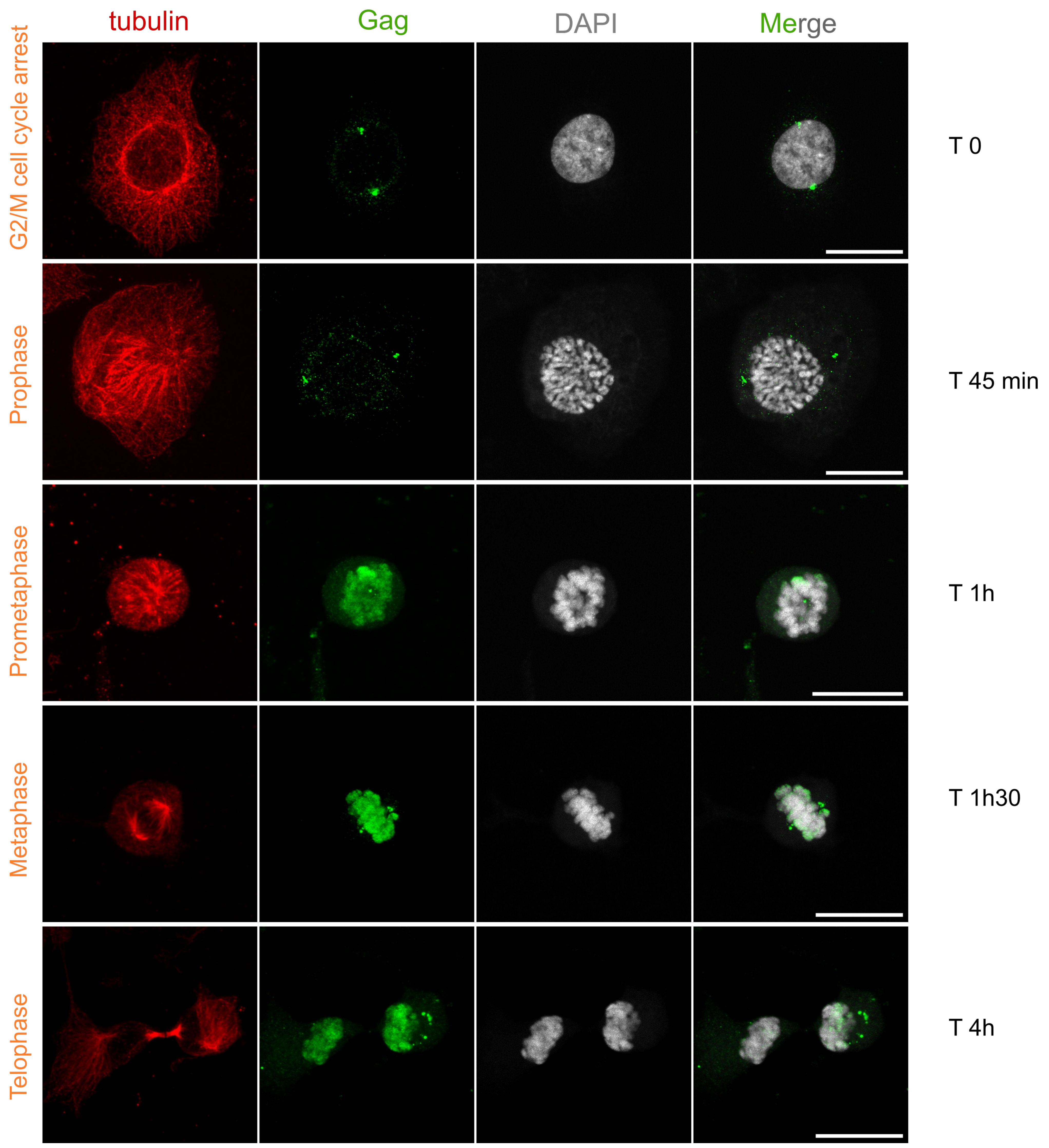

Figure S1 - Optimization of cell cycle synchronization. HT1080 cells were treated with 20 µM RO-3306, transduced with PFV particles and fixed at different time points post RO-3306 release. Mitotic phases and their corresponding time points were identified via immunostaining of tubulin (red) and PFV Gag (green). Scale: 20 µm.

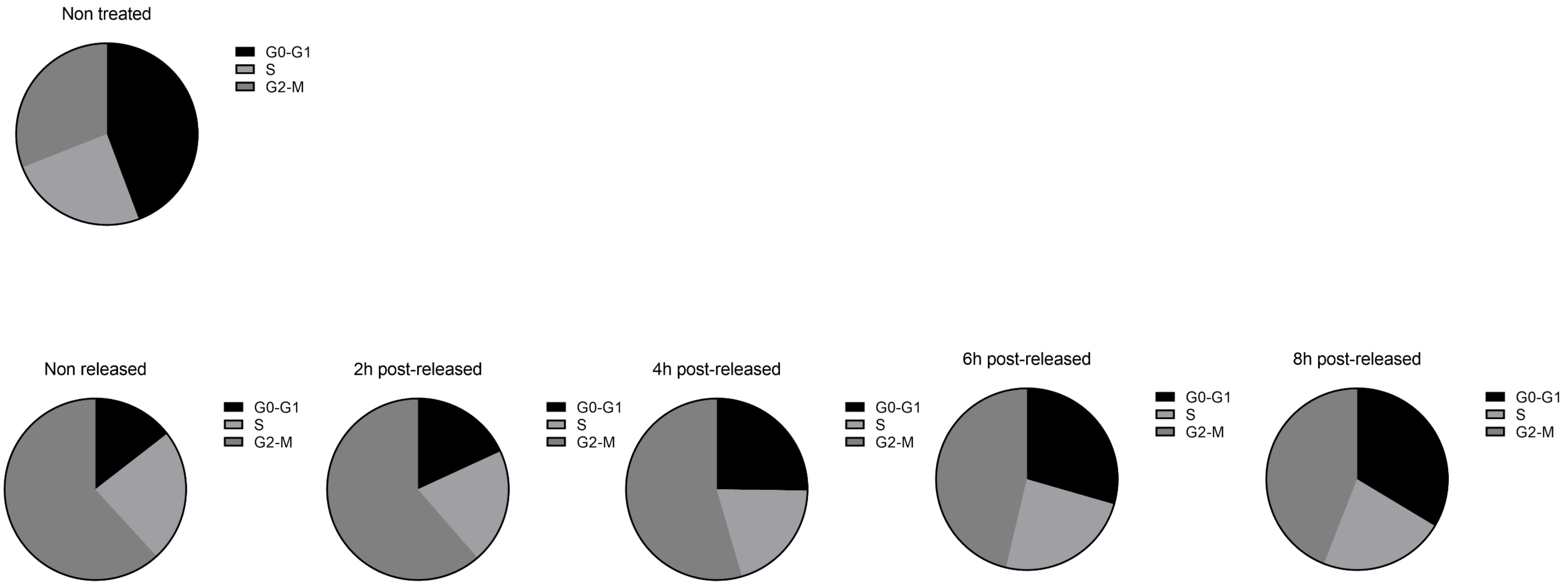

Figure S2 - Cell cycle recovery of HT1080 cells after RO-3306 treatment. HT1080 cells were either treated with RO-3306 or not, and their DNA content was monitored at different time points using propidium iodide dye staining and FACS.

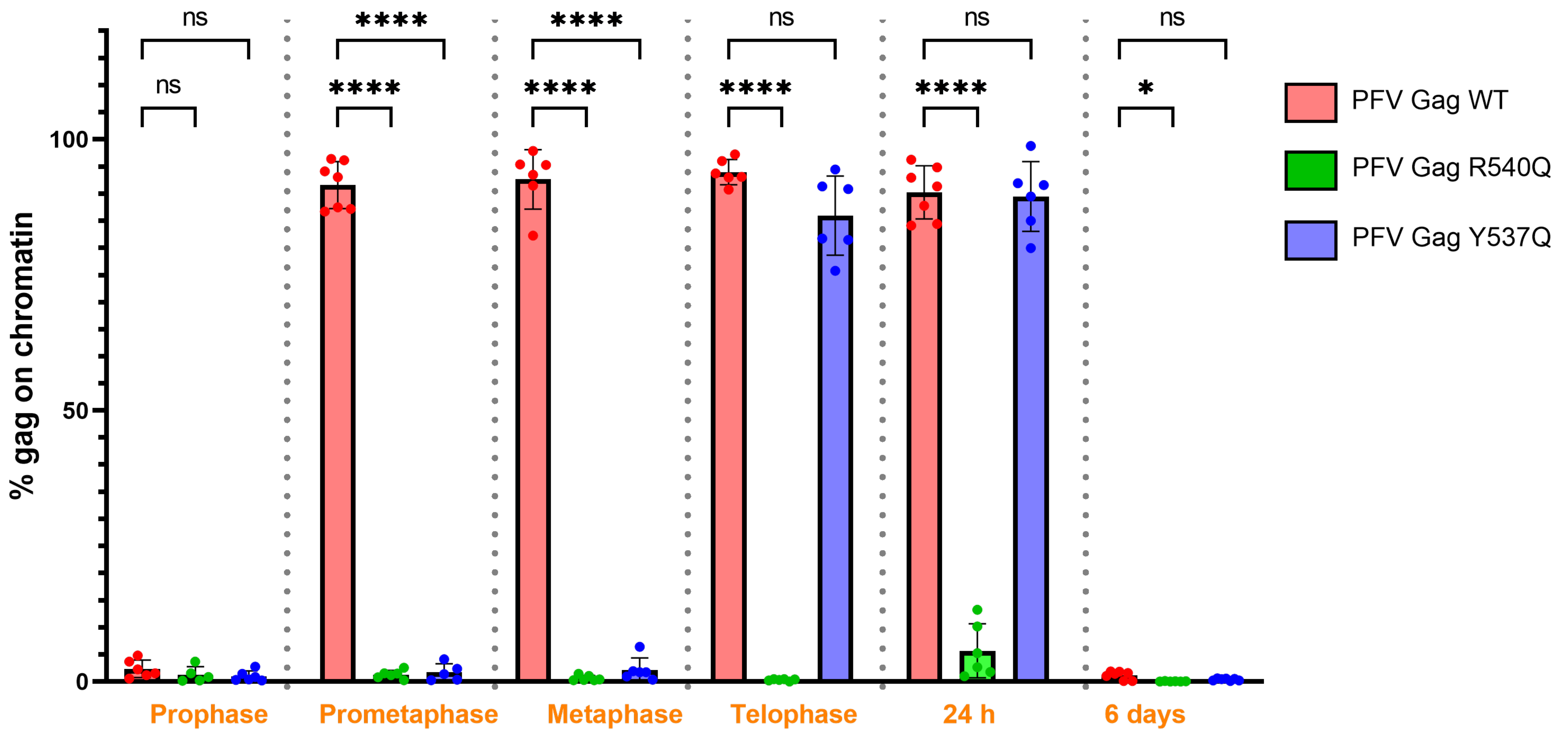

Figure S3 - Quantification of Gag-chromatin binding as a function of mitotic phase. The percentage of chromatin surface recovered by Gag was quantified for each mitotic phase analyzed in Fig. 3. The mean of a minimum of 5 cells per condition was represented, with their corresponding standard deviation. Statistical analysis was performed using the two way ANOVA with the Tukey's multiple comparisons tests (* $p < 0.05$; **** $p < 0.0001$).

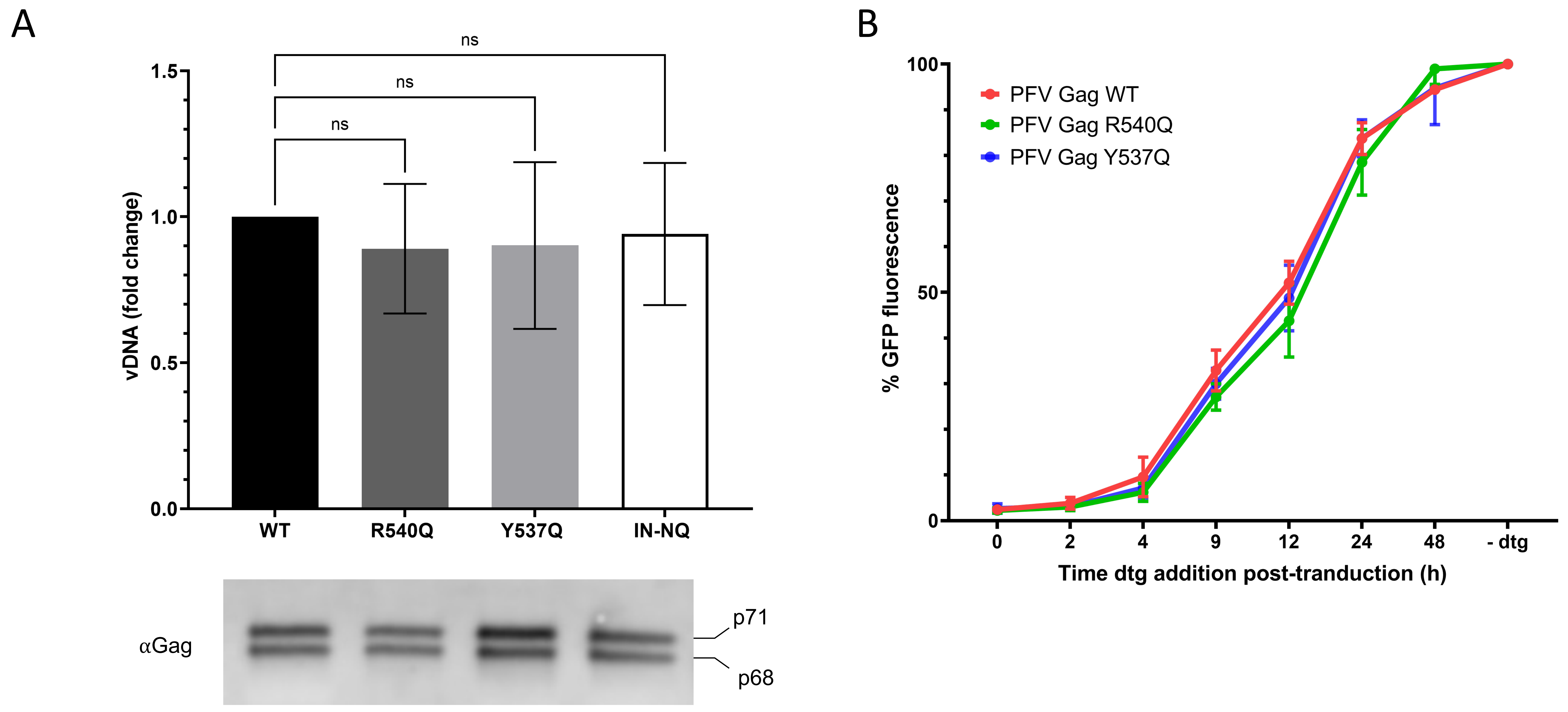

Figure S4 - PFV particle loading controls. (A) Quantitative PCR of viral DNA used for transduction of HT1080 cells with PFV vector particles carrying WT, R540Q or Y537Q Gag with WT IN, or the WT Gag containing catalytically inert integrase (IN-NQ). Cells were harvested 1 h post-infection and the viral DNA was extracted and subjected to qPCR. The WT condition was set to 1 and results are expressed as comparative fold change. Immunoblots detecting Gag precursors (p71 and p68), which were used to control for viral input. The mean of three independent experiments is plotted. (B) Integration kinetics of WT and CBS mutants viruses monitored by inhibition of viral integration after dolutegravir (DTG) addition at different time points post-infection. Values without DTG for each condition were arbitrarily set to 100%. Relative means and standard deviations from three independent experiments are shown.

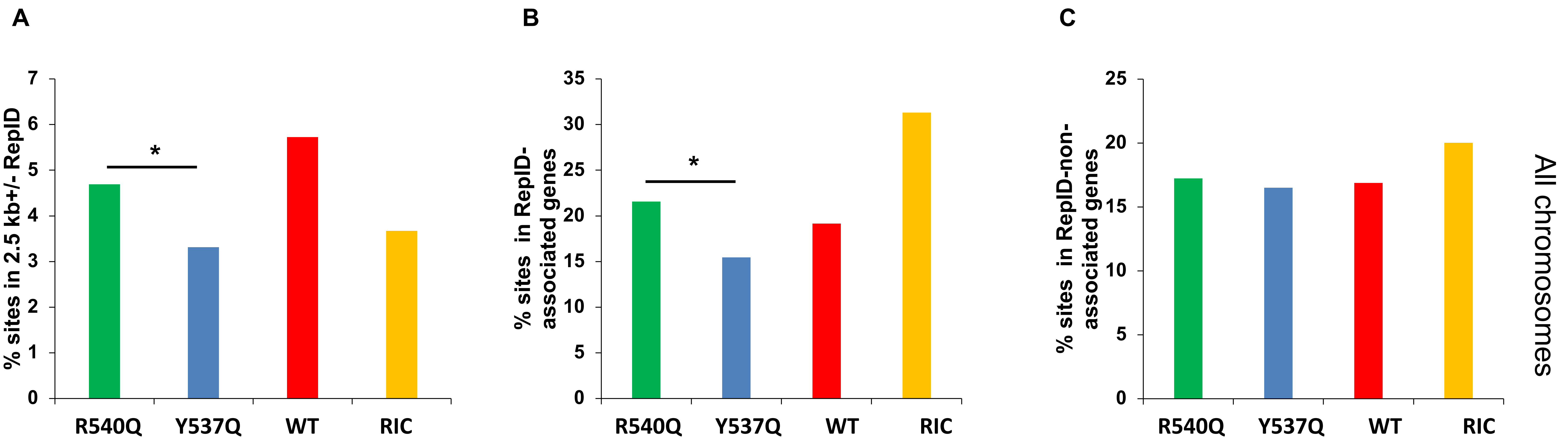

Figure S5 - Correlation between replication-initiation determinant protein (RepID) binding sites and PFV integration. (A) PFV integration sites (%) within +/- 2.5 kb of RepID ChIP-Seq sites, (B) RepID-ChIP-Seq associated genes and (C) non-associated genes ($p < 0.05$ between R540Q and Y537Q; Fisher's exact test).

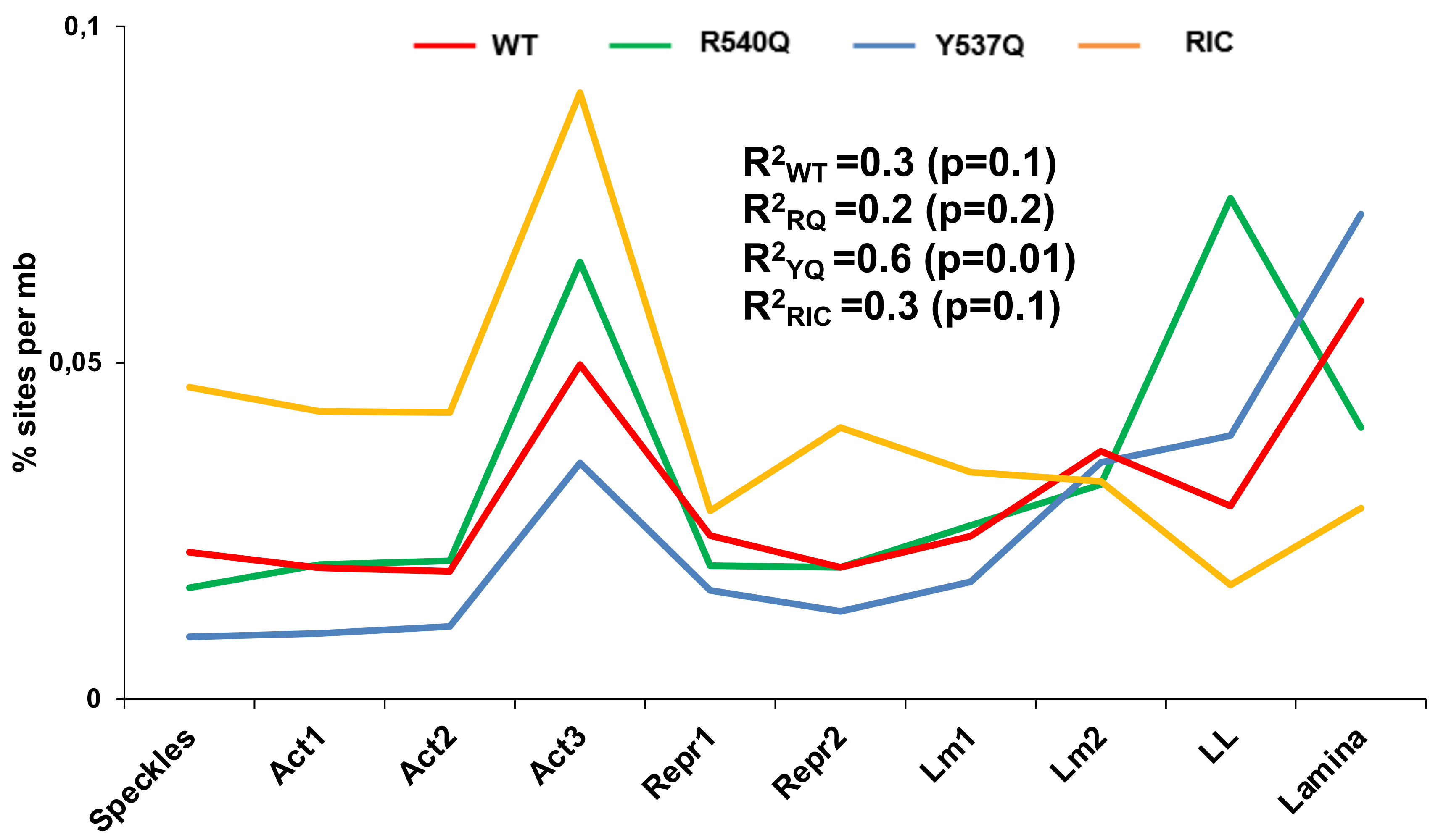

Figure S6 – Distribution of PFV integration sites with respect to SPIN states. PFV integration sites (%) per Mb across 10 Spatial Position Inference of the Nuclear genome SPIN states (X-axis) were calculated for WT, R540Q (RQ) and Y537Q (YQ) PFVs along with a random integration control (RIC). Coefficients of determination (R^2) between integration % (Y-axis) and SPIN states are shown (calculated in Excel and square root of R^2 was used to calculate p values at https://www.socscistatistics.com/pvalues/pearsondistribution.aspx). Act1, Interior Active 1; Act2, Interior Active 2; Act3, Interior Active 3; Repr1, Interior Repressive 1; Repr2, Interior Repressive 2; Lm1, Near Lamina 1; Lm2, Near Lamina 2; LL, Lamina-Like.

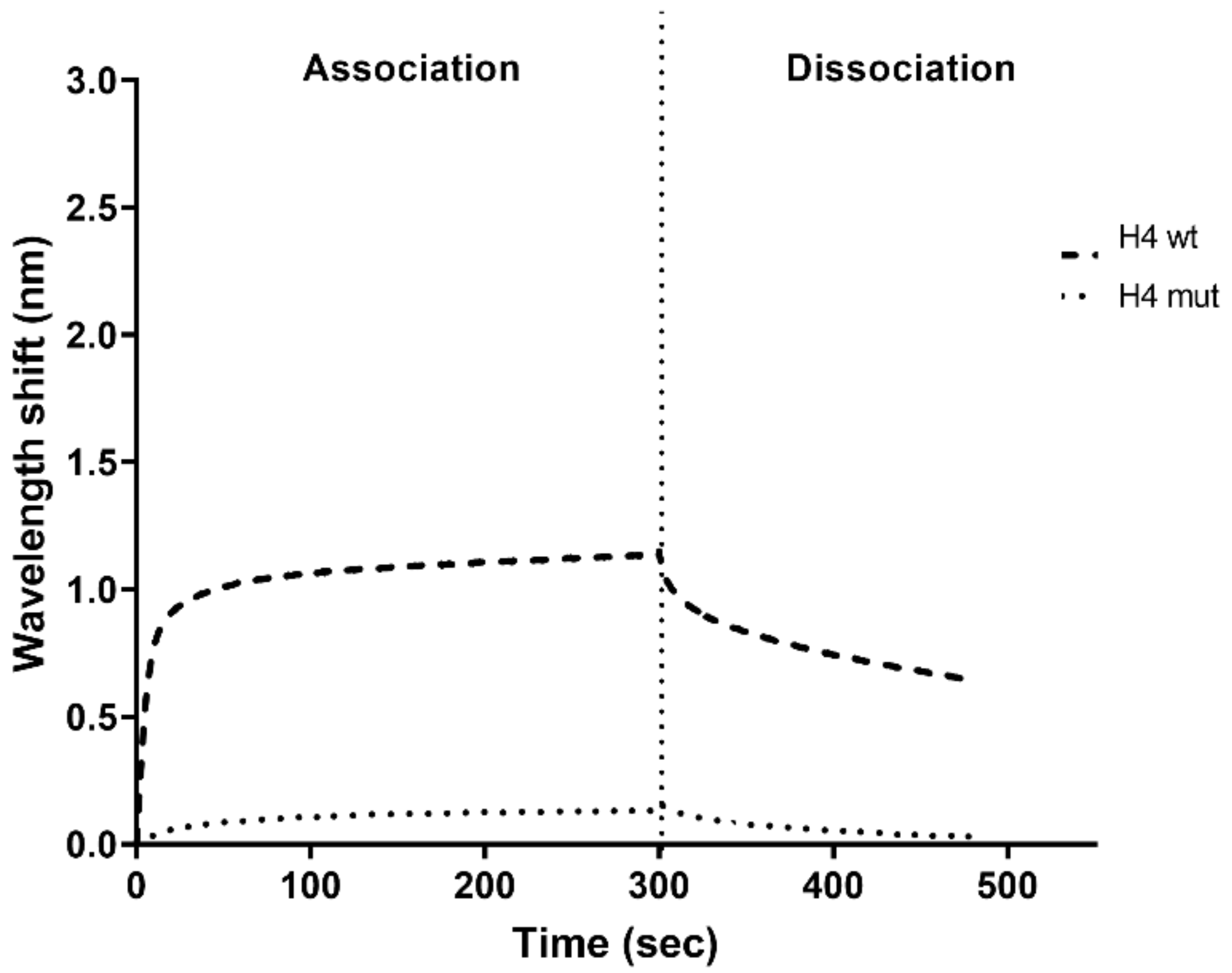

Figure S7 - H4 tail-NCP interaction controls. Bio-Layer interferometry (BLI) sensorgram of free nucleosomes binding to immobilized WT H4 tail (residues 2-24) (dashed line) or H4 mut (triple alanine substitution) (dotted line). The binding intensity (nm) is normalized with a condition without NCP. The mean of 2 independent experiments is plotted.
